# Predictors of tuberculosis treatment outcomes in Antananarivo: a retrospective cohort study

**DOI:** 10.11604/pamj.2023.46.104.41514

**Published:** 2023-12-13

**Authors:** Sedera Radoniaina Rakotondrasoa, Antso Hasina Raherinandrasana, Norotiana Ramanarivo, Tantely Jenny Ramontalambo, Zina Antonio Randriananahirana, Lantonirina Ravaoarisoa, Julio Rakotonirina

**Affiliations:** 1 Faculty of Medicine of Antananarivo, Antananarivo, Madagascar,; 2 National Institute of Public and Community Health (INSPC), Antananarivo, Madagascar,; 3 Hospital for Care and Public Health of Analakely (CHUSSPA), Antananarivo, Madagascar

**Keywords:** Predictors, Madagascar, outpatients, treatment outcomes, tuberculosis

## Abstract

**Introduction:**

Tuberculosis (TB) is a global public health issue, affecting Africa and Madagascar. Adverse outcomes following ineffective treatment are common. Previous studies conducted in similar settings have not adequately accounted for confounding factors. The objective of this study is to identify predictive factors that are associated with tuberculosis treatment outcomes in Madagascar.

**Methods:**

a retrospective cohort study was conducted using registries of 628 outpatients with tuberculosis at the Analakely Hospital (CHUSSPA) in 2019. Univariate and multivariate logistic regression analyses were performed.

**Results:**

the study included 628 patients with a mean age of 37.19 ± 15.86 years and a sex ratio of 1.57. These patients were followed up for a total of 2886 person-months. Out of the 628, 517 achieved treatment success, while 31 patients died and 31 discontinued their treatment. The rates of treatment success, death, failure, and default were 82.3%, 4.9%, 0.2%, and 8.3% respectively. Female gender was found to be a predictor of treatment success area of responsibility adjusted odds ratio(AOR 1.67 [1.07-2.66]; p=0.026). Smear-negative pulmonary tuberculosis (SNPTB) was associated with a lower likelihood of treatment success (AOR 0.38 [0.23-0.65]; p<0.001) and was a common factor for default (AOR 3.17 [1.60-6.21]; p=0.001) and death (AOR=8.03 [3.01-23.72; p<0.001]). Extra-pulmonary TB was identified as a predictor of death (AOR 5.15 [1.99-14.95]; p=0.001).

**Conclusion:**

the tuberculosis treatment indicators in this center have not yet met national and global targets. It is necessary to focus on early diagnosis, improving education, and implementing rigorous follow-up procedures for patients at high risk of adverse outcomes (SNPTB and extra-pulmonary tuberculosis(EPTB)

## Introduction

Tuberculosis affected approximately 10.6 million people in 2021 [1]. It is one of the six leading causes of death and the leading infectious cause of death, surpassing HIV/AIDS. The key to controlling this disease is standardized short-term treatment with chemotherapy regimens lasting at least of 6 to 8 months [2]. In 2020, global data showed an overall treatment success rate of 86% [1]. However, compliance issues have always been a significant factor in treatment failure and negative outcomes. Patients who are lost to follow-up and non-compliant are exposed to clinical deterioration, complications, tuberculosis recurrence, treatment failure, and even death [3,4]. Additionally, these patients can spread the disease within their communities, as they remain contagious [5]. The treatment outcomes reflect the effectiveness of tuberculosis control efforts. Africa comes in second place among the continents most affected by the disease, just after South-East Asia [1]. The treatment success rate in the African region is still low compared with the WHO target, with a rate of only 79% in 2019 [6]. In Madagascar, tuberculosis remains a major public health concern. The incidence rate in 2021 was estimated at 233 (149 - 336) cases per 100,000 inhabitants for all forms of the disease, and the mortality rate was estimated at 48 per 100,000 [7]. Unfortunately, the treatment success rate for new and relapsed cases was 83% in 2019, falling short of the global target of 90% [1]. Previous studies have mostly focused on examining the factors associated with different treatment outcomes separately or by combining them into a single variable [8,9]. A meta-analysis conducted in Africa revealed significant variations in these factors between African countries [6]. The most commonly cited factors linked to unsuccessful TB treatment outcomes are HIV co-infection and retreatment [6]. However, research on the factors influencing treatment outcomes in Madagascar is scarce and limited in scope, often failing to adequately account for potential confounders [10,11]. Therefore, it is necessary to conduct studies that address these methodological limitations to enhance our understanding of these factors within the context of tuberculosis treatment in Madagascar. Identifying these factors accurately could help health workers and authorities make informed decisions, and plan and implement effective interventions to control the spread of tuberculosis. The End TB strategy of the World Health Organization emphasizes integrated, patient-centered care and prevention, robust policies and supportive systems, as well as intensified research and innovation [12]. Identifying shared factors in treatment outcomes could help advance these strategies by providing a broader, comprehensive understanding of the individual or contextual factors that characterize the patient. The objective of this paper is to determine predictive factors that are associated with TB treatment outcomes in the town of Antananarivo.

## Methods

The present study consisted of a retrospective cohort study based on secondary data analysis. It was carried out at the Tuberculosis Diagnosis and Treatment unit of the Analakely Hospital (CHUSSPA) in Antananarivo, the capital of Madagascar.

**Data source:** the data were collected from registries of tuberculosis patients who were diagnosed and treated at the study site. These data were gathered as part of a systematic ambulatory follow-up of treated patients. Follow-ups were spread over a six-month period, which corresponds to the length of a full treatment course. All patients were followed up using a similar procedure. However, it is important to note that these data have limitations in that only a limited number of variables are available. Furthermore, the sample constituted by the data might not provide a representative sample of Antananarivo due to its single-center approach. Additionally, complicated cases were systematically transferred to a specialized pneumology hospital, resulting in a lack of data on the therapeutic results of these transferred patients. Despite these limitations, we believe that the data could yield interesting findings if external consistency is ensured. Moreover, the object of the initial data collection is consistent with the current study. It is worth mentioning that these data were kept strictly confidential, with access restricted to treatment center staff. Approval from the head of the facility was obtained before using the data.

**Study population:** this study used the data of all patients who had been diagnosed and treated at this facility from January 1^st^ to December 31^st^, 2019. All patients for whom treatment outcome data were available were exhaustively selected. The study consisted of a dynamic cohort, in which subjects were included at different dates but were followed up over a theoretical 6-month period, unless they had died, were lost to follow-up, or experienced a therapeutic failure.

**Sample size:** the minimum sample size of 602 was calculated using the formula of Fleiss JL *et al*. [13], utilizing the OpenEpi website [14]. This calculation was based on an alpha level of 5%, and a beta level of 20%.

**Variables:** the dependent variables were represented by treatment success, treatment failure, default, and death during treatment. The criteria used for determining therapeutic success and failure were based on the revised WHO standard definition in 2021 [15]. Treatment success refers to all patients declared cured and patients who have not yet completed the final sputum examination, but who have completed treatment. These two categories have been grouped under the therapeutic success category in accordance with the WHO definitions update, where completed treatment is defined as a tuberculosis patient who has completed the treatment without evidence of failure, but without records showing that sputum smear or culture results in the last month of treatment and on at least one previous occasion were negative, either because tests were not conducted or because results are not available [15]. Failure is defined as the presence of positive sputum at the 5^th^ month or later in consecutive treatments. Drop-out or default status corresponds to 2 months of interrupted treatment. The explanatory variables included gender, age, area of residence (rural/urban), occupation (salaried/unsalaried), tuberculosis clinical form (smear-positive pulmonary tuberculosis or SPPTB, smear-negative pulmonary tuberculosis or SNPTB, extra-pulmonary tuberculosis or EPTB), and patient treatment status (new case/retreatment).

**Data extraction and processing:** the relevant variables were extracted from patient registries and manually inputted into MS Excel®. We carefully reviewed the data entries for consistency. Any observations with missing data were removed if they accounted for less than 10% of the total for each relevant variable. Quantitative variables were manually discretized using the threshold most commonly used in the literature, to ensure that our findings would be comparable.

**Data analysis:** the data was analyzed using R® software. First, descriptive analysis was performed, estimating frequencies and percentages for qualitative variables, and means with their standard deviations for quantitative variables. Bivariate analyses with simple logistic regression were then carried out to identify factors associated with treatment outcomes under univariate analysis. Variables with p-values below 0.2 were included in the multivariate logistic regression model. The best model was chosen using an approach that involved the stepwise function and Aikake's information criteria. To check the quality of the model, a goodness-of-fit test and a model specification test (linktest) were applied. The significance threshold for the multivariate analysis was set at p ≤ 0.05. Unfortunately, due to the small number of available cases, it was not possible to determine the predictor of treatment failure. Patients who were lost to follow-up were included in the final analysis. However, to address potential bias, we conducted an additional analysis to identify predictive factors for treatment default.

**Ethical consideration:** approval was obtained from the Ethics Committee on Biomedical Research in Madagascar, as well as from the heads of the facility under study, before initiating the study. The data was meticulously kept confidential throughout the research process and was protected using passwords. Observations remained anonymous and no patient information was disclosed to maintain privacy.

## Results

**Figure 1:**
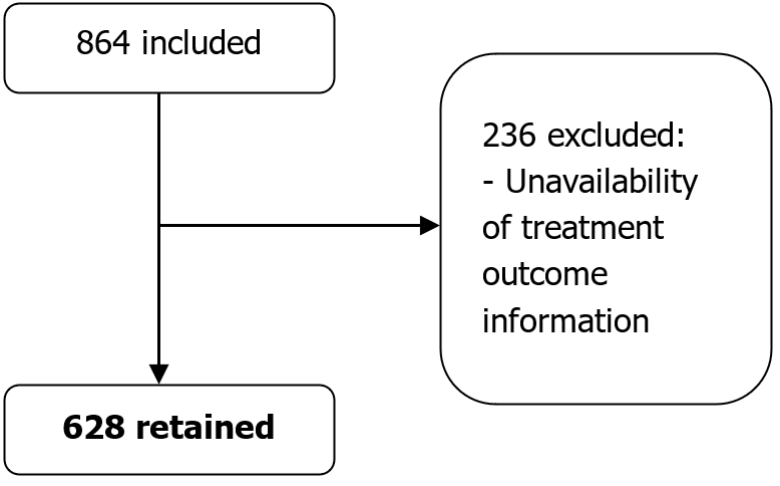
participant selection flow chart

A total of 628 patients, with an average age of 37.19 years and a standard deviation of 15.86 years, were included in the analysis. Figure 1 illustrates the changes in the sample size during the patient screening process. Table 1 presents the demographic characteristics of the sample. The majority of participants are male (61.1%), aged 15 to 29 years (37.3%), unemployed (56.5%), and residing in rural areas (80.7%). Our findings revealed that among the 628 patients, therapeutic success was the primary outcome. Female gender and SNPTB were identified as predictors of therapeutic success. Smear-negative pulmonary tuberculosis and EPTB were identified as predictors of death. Additionally, SNPTB was the only predictor of treatment default.

**Table 1:** sample characteristics

Variables	N=628	%
**Gender**		
Female	244	38.9
Male	384	61.1
**Age**		
<15 years	13	2.1
15-29 years	234	37.3
30-44 years	179	28.5
45- 59 years	142	22.6
60 and over	60	9.6
**Profession**		
Not employed	273	43.5
Employed	355	56.5
**Residence**		
Rural	121	19.3
Urban	507	80.7
**Clinical form**		
EPTB	169	26.9
SNPTB	99	15.8
SPPTB	360	57.3
**Treatment status**		
New cases	579	92.2
Retreatment	49	7.8

SPPTB: smear-positive pulmonary tuberculosis, SNPTB: smear-negative pulmonary tuberculosis, EPTB: extra-pulmonary tuberculosis

**Follow-up and treatment outcomes:** overall follow-up time was 2,886 person-months (mean 5.01±0.03 months, maximum 11 months). During this follow-up period, 517 patients achieved therapeutic success, while 31 patients died and 31 patients discontinued their treatment. Therapeutic success was the most frequent outcome of the study, representing 82.3% of cases. The death rate observed was 4.9%, with a failure rate of 0.2% and a default rate of 8.3%.

**Results of association measurement:** in multivariate analysis, being female (AOR 1.67 [1.07-2.66], p=0.026) and having SNPTB (AOR 0.38 [0.23-0.65], p<0.001) were identified as predictors of therapeutic success (Table 2). Specifically, women have a 1.67 times higher likelihood of treatment success compared to men. Smear-negative pulmonary tuberculosis patients, on the other hand, have a 62% lower likelihood of treatment success compared to EPTB patients. Smear-negative pulmonary tuberculosis (AOR 8.03 [3.01-23.72], p<0.001) and EPTB (AOR 5.15 [1.99-14.95]; p=0.001) were identified as predictors of death (Table 3). Specifically, patients with SNPTB were 8.03 times more likely to die than SPPTB patients, while those with EPTB were 5.15 times more likely to die, all else being equal. Smear-negative pulmonary tuberculosis (AOR 3.17 [1.60-6.21]; p=0.001) was the only predictor of treatment default that was identified. Patients with SNPTB were 3.17 times more likely to discontinue their tuberculosis treatment compared to those with SPPTB.

**Table 2:** predictors of successful treatment among CHUSSPA TB patients in 2019 - Univariate and multivariate logistic regression analysis

Variables	Treatment success		Univariate	Multivariate
	Yes	No	OR (CI 95 %; p)	AOR (CI 95%; p)
	n=517 (%)	n=111 (%)		
**Gender**				
Male	305 (79.43)	79 (20.57)	1	-
Female	212 (86.89)	32 (13.11)	1.72 (1.11-2.71; p=0.018)	1.67 (1.07-2.66; p=0.026)
**Age**				
<15 years	10 (76.92)	3 (23.08)	1	-
15-29 years	203 (86.75)	31 (13.25)	1.96 (0.42-6.85; p=0.325)	-
30-44 years	142 (79.33)	37 (20.67)	1.15 (0.25-3.99; p=0.837)	-
45- 59 years	116 (81.69)	26 (18.31)	1.34 (0.29-4.74; p=0.674)	-
60 and over	46 (76.67)	14 (23.33)	0.99 (0.20-3.77; p=0.984)	-
**Profession**				
Not employed	223 (81.68)	50 (18.32)	1	-
Employed	294 (82.82)	61 (17.18)	1.08 (0.71-1.63; p=0.712)	-
**Residence**				
Rural	101 (83.47)	20 (16.53)	1	-
Urban	416 (82.05)	91 (17.95)	0.91 (0.52-1.51; p=0.713)	-
**Clinical form**				
SPPTB	310 (86.11)	50 (13.89)	1	-
SNPTB	69 (69.7)	30 (30.3)	0.37 (0.22-0.63; p<0.001)	0.38 (0.23-0.65; p<0.001)
EPTB	138 (81.66)	31 (18.34)	0.72 (0.44-1.18; p=0.186)	0.71 (0.43-1.16; p=0.167)
**Treatment status**				
New cases	477 (82.38)	102 (17.62)	1	-
Retreatment	40 (81.63)	9 (18.37)	0.95 (0.47-2.14; p=0.895)	-

OR: odds ratio; AOR: adjusted Odds ratio; CI: confidence interval; SPPTB: smear-positive pulmonary tuberculosis, SNPT: smear-negative pulmonary tuberculosis, EPTB: extra-pulmonary tuberculosis

**Table 3:** predictors of death during treatment among CHUSSPA TB patients in 2019 - Univariate and multivariate logistic regression analysis

Variables	Death		Univariate	Multivariate
	Yes	No	OR (CI 95 %; p)	AOR (CI 95 %; p)
	n=31 (%)	N=597 (%)		
**Gender**				
Male	24 (6.25)	360 (93.75)	Ref	-
Female	7 (2.87)	237 (97.13)	0.44 (0.17-0.99; p=0.063)	0.47 (0.18-1.07; p=0.089)
**Age**				
<15 years	0 (0)	13 (100)	Ref	-
15-29 years	2 (0.85)	232 (99.15)	3.6E5 (0.00 -NA; p=0.991)	-
30-44 years	10 (5.59)	169 (94.41)	2.5E6 (8.5E144-5,4E168; p=0.989)	-
45- 59 years	9 (6.34)	133 (93.66)	2.9E6 (0.00 -NA; p=0.989)	-
60 and over	10 (16.67)	50 (83.33)	8.5E6 (0.00 -NA; p=0.988)	-
**Profession**				
Not employed	16 (5.86)	257 (94.14)	Ref	-
Employed	15 (4.23)	340 (95.77)	0.71 (0.34-1.47; p=0.350)	-
**Residence**				
Rural	3 (2.48)	118 (97.52)	Ref	-
Urban	28 (5.52)	479 (94.48)	2.30 (0.80-9.73; p=0.177)	2.43 (0.83-10.39. p=0.155)
**Clinical form**				
SPPTB	6 (1.67)	354 (98.33)	Ref	-
SNPTB	12 (12.12)	87 (87.88)	8.14 (3.07-23.94; p<0.001)	8.03 (3.01-23.72; p<0.001)
EPTB	13 (7.69)	156 (92.31)	4.92 (1.91-14.21; p=0.002)	5.15 (1.99-14.95; p=0.001)
**Treatment status**				
New cases	29 (5.01)	550 (94.99)	Ref	-
Retreatment	2 (4.08)	47 (95.92)	0.81 (0.13-2.80; p=0.774)	-

OR: podds ratio; AOR: adjusted Odds ratio; CI: confidence interval; SPPTB: smear-positive pulmonary tuberculosis, SNPTB: smear-negative pulmonary tuberculosis, EPTB: extra-pulmonary tuberculosis

## Discussion

This study aimed to identify factors that can predict the outcomes of TB treatment. This study made a valuable contribution to the existing literature by improving the methodology used in similar studies conducted in Madagascar, with a confirmation view. However, it is important to acknowledge that there may still be some bias present, as the available variables in the source database were limited to ensure that all potential confounders were included in the statistical model with certainty. Nevertheless, the model specification test confirmed an acceptable quality of the final statistical model, which convinced us to proceed with this study. It also provides a comprehensive view of adverse outcome predictors by identifying their common factors within a single cohort. Thus, the results of this study showed that the rates of treatment success, death, failure, and default did not meet national targets. Being female was associated with a higher likelihood of treatment success. Smear-negative pulmonary tuberculosis was found to be a negative factor for success and a common factor for default and death. Extra-pulmonary tuberculosis was linked to an increased risk of death.

**Therapeutic success:** the therapeutic success rate identified in this study was 82.3%, which is lower than the national and global targets of 85% and 90%, respectively [16,17]. It is also lower than the overall African therapeutic success rate of 86% reported in 2020 [1]. Tiaray *et al*. also found a higher success rate (89%) in a different treatment center in the same city [11]. The female gender was found to be a favorable factor for therapeutic success. This finding contradicts with the majority of studies conducted in Africa, which have shown that gender is not associated with therapeutic success [6]. However, it is worth noting that some authors have discovered that men were less likely to adhere to treatment [18]. Our result may be influenced by the fact that men were over-represented in our sample (Table 1). Smear-negative pulmonary tuberculosis was found to be a predictor of unsuccessful treatment, in line with previous studies [19]. This form of TB is often associated with adverse treatment outcomes and lower adherence due to its atypical presentation, challenging diagnosis, and frequent co-morbidities [20,21]. These findings highlight the importance of early diagnosis and effective treatment for patients with SNPTB to prevent disease transmission, complications, and default. Furthermore, these results open up new perspectives for further research to elucidate factors linked to SNPTB that influence its prognosis. It also offers a new avenue to evaluate the impact of interventions aimed at improving treatment adherence in patients with SNPTB.

**Death during treatment:** this study revealed a death rate of 4.9%, which is higher than that reported in other studies conducted in other low-income countries [22,23]. One possible reason for the high rate could be the prevalence of SNPTB and EPTB, which were identified as risk factors of death in this study (Table 3). These findings align with those of Djouma *et al*. [20] and Alobu I *et al*. [24]. Both of these forms of tuberculosis are strongly linked to mortality and treatment default, likely due to the commonly associated co-morbidities [21,24]. Extrapulmonary forms of tuberculosis are typically paucibacillary and negative on direct sputum examination, requiring additional tests such as PCR, culture, and histology to confirm the diagnosis. Treatment delays can therefore lead to mortality. In cases of TB/HIV co-infections, the SNPTB form is commonly observed in individuals with advanced immunosuppression [25,26]. However, diagnosing this form is challenging, as many patients are misclassified as SNPTB based solely on clinical data [27] and the results of direct sputum examination. In some instances, these cases of mortality may have a non-tuberculous origin. It would, therefore, be crucial to confirm these cases using the Gene Xpert MTB/RIF test for more reliable results [28]. However, this test is often expensive and financially inaccessible to patients. Clinicians should recognize the significance of effectively detecting and treating SNPTB and extrapulmonary forms of tuberculosis to minimize the risk of death in these patients. Targeted screening programs and additional support for treatment adherence are essential for managing these high-risk individuals. Future research should delve into the specific causes of death in TB patients, particularly in cases of HIV co-infection. Exploring funding options for diagnostic tests to enhance accessibility in these settings is crucial. Additionally, assessing the effectiveness of targeted screening programs for these high-risk patients, including the impact of the Gene Xpert MTB/RIF test on survival and TB transmission in the community is essential. Further investigation into interventions to enhance treatment adherence and reduce mortality is also warranted.

**Therapeutic failure:** the failure rate assessment in this series revealed a low rate of 0.2%, which is lower than the rates reported in studies by Cardoso [23] and Adane K *et al*. [22], where rates of 5% and 1.6%, respectively, were reported. One possible reason for this difference is the exclusion of a substantial number of patients from this study who might have been classified as failure cases. Unfortunately, analyzing the relationship between failure and study parameters was not possible due to the limited number of included therapeutic failure cases (n=1). Further studies with a larger patient cohort and an extended duration would be necessary.

**Treatment default:** the default rate found in this study was 8.3% (Figure 1), which has not yet reached the national program target (<7%) [16]. Additionaly, it is higher compared to the findings reported by Adane K [22] and Rakotoson JL [10], who found rates of 1.4% and 4.5%, respectively. This high rate suggests the necessity to reinforce strategies aimed at improving adherence. The only default factor identified in this study was SNPTB, aligning with the findings of Alobu I *et al*. [24] and Pefura Yone *et al*. [29]. These authors reported that in low-income countries, many patients discontinue treatment during the initial phase (first 2 months) due to the direct and indirect costs of care and the resolution of symptoms [24]. Smear-negative pulmonary tuberculosis is characterized by an attenuation of symptoms [27], which may be interpreted by patients as a lack of severity, potentially diminishing their motivation, as well as that of their families, to pursue treatment. To mitigate this, intensive patient education, particularly during the 2^nd^ and 4^th^ months of treatment, along with financial support, is needed. Future research should assess the implementation and impact of interventions to improve adherence on default rates and TB treatment outcomes. Additionally, socio-cultural, economic, and behavioral factors influencing tuberculosis treatment default, especially in patients with SNPTB, should be thoroughly investigated.

**Study limitations:** it is worth mentioning some limitations of this study that could affect its interpretation and generalizability. Firstly, it is representative of a single tuberculosis diagnostic and treatment center, focusing on outpatient cases. Therefore, the findings may not fully capture the diversity of treatment centers in Antananarivo, some of which admit complicated cases. Moreover, due to the restricted availability of data and the retrospective nature of the study, certain relevant clinical indicators, such as germ eradication, drug resistance, and patient quality of life, could not be examined. Socio-economic, cultural, and behavioral factors likely to influence treatment adherence and tuberculosis risk exposure were not taken into account. The lack of variables could also have led to the omission of potential confounding factors in the final statistical model. However, the model specification was checked to ensure acceptable completeness, providing reliable and valid estimates of the association measure parameters. Additionally, the reliability of these results was verified for consistency with other studies. The interpretation of the results on death should be relativized, as the cause of these deaths could be of non-tuberculosis origin. Yet, neither the diagnoses of death nor the causes of these deaths were mentioned in the patient registers. Nevertheless, a certain external consistency of these results was identified in this study.

## Conclusion

This study aimed to identify factors associated with different therapeutic outcomes for tuberculosis. Despite its limitations, particularly in terms of variable availability, it has the potential to contribute to existing knowledge by confirming some previous findings in the Malagasy context. It also offers a more synthetic view of the predictors of unfavorable outcomes by identifying their common factors within the same cohort. The study showed that therapeutic outcome indicators at the study site were close to the national program target but were not satisfactory, particularly for default and death. Smear-negative pulmonary tuberculosis was a common factor in unfavorable outcomes. These findings confirm the results of previous studies in the same context and certain low-income countries. They imply that clinicians should ensure early and correct screening, education, effective treatment, and follow-up of high-risk areas for adverse outcomes, particularly SNPTB and extrapulmonary tuberculosis. Improving access to the Gene Xpert MTB/RIF test should be a strategic priority in the program to combat this disease. Future research paths have been identified, including multicentric studies, explorations of the exact causes of death in tuberculosis patients, studies assessing the effectiveness of targeted screening programs, and the implementation and impact of interventions aimed at improving therapeutic compliance.

### What is known about this topic

- *A meta-analysis of factors associated with tuberculosis treatment outcomes in Africa found that these factors differed considerably between African countries; the most frequently cited factors associated with unsuccessful TB treatment outcomes in the literature were HIV co-infection and retreatment;*
- *The majority of prior studies in the context of low-income countries have often focused on studying adverse outcomes separately or recoding various therapeutic outcomes into a single variable, hindering the identification of their common factors;*
- *Additionally, in the context of Madagascar, previous studies have not sufficiently controlled for potential confounding factors, limiting the evidence on these factors*.

### What this study adds

- *This study contributes to existing knowledge by validating previous findings on factors influencing tuberculosis treatment in the Malagasy context; it offers a comprehensive analysis of adverse outcome predictors by identifying common factors within the same cohort;*
- *The study revealed that therapeutic outcome indicators at the study site were near the national program target but fell short of satisfaction, especially concerning default and mortality rates;*
- *Smear-negative pulmonary tuberculosis emerged as a common factor in unfavorable outcomes; additionally, extra-pulmonary tuberculosis was found to be linked to an increased risk of death; finally, the study indicated that being female was associated with a higher likelihood of treatment success*.
